# Propylthiouracil-Induced Antineutrophil Cytoplasmic Antibody-Associated Vasculitis after COVID-19 Vaccination

**DOI:** 10.3390/vaccines9080842

**Published:** 2021-07-31

**Authors:** Saki Okuda, Yasuaki Hirooka, Masafumi Sugiyama

**Affiliations:** Department of Rheumatology, Kindai University Nara Hospital, 1248-1, Otodacho, Ikoma 630-0293, Japan; 2095C8@med.kindai.ac.jp (S.O.); m-sugi@med.kindai.ac.jp (M.S.)

**Keywords:** propylthiouracil, ANCA-associated vasculitis, relapsing polychondritis, COVID-19 vaccine

## Abstract

We report the case of a patient who developed antineutrophil cytoplasmic antibody (ANCA)-associated vasculitis (AAV) after receiving the coronavirus disease 2019 (COVID-19) vaccine BNT162b (Pfizer–BioNTech). A 37-year-old Japanese woman had been taking propylthiouracil for Graves’ disease. She had erythema on her forearm on the 12th day after receiving the first dose of the vaccine, fever on the 13th day, and redness and swelling of her left auricle on the 25th day. Her serum myeloperoxidase-ANCA and proteinase 3-ANCA levels, which were negative before the Graves’ disease treatment, were elevated. She had unilateral auricular symptoms but no other typical relapsing polychondritis findings. She was diagnosed with propylthiouracil-induced AAV. She was treated with oral glucocorticoids, and her symptoms improved. Propylthiouracil is considered to be the main cause of the onset of AAV in this case, but it cannot be ruled out that BNT162b may have had some effect on the onset of the disease. Although the development of propylthiouracil-induced AAV in this case may have been incidental and unrelated to the vaccination, this report provides important data for evaluating the safety of the vaccine.

## 1. Introduction

Coronavirus disease 2019 (COVID-19), caused by severe acute respiratory syndrome coronavirus 2 (SARS-CoV-2) and first detected in the city of Wuhan in China’s Hubei Province, has become a global pandemic. To date, more than 178 million people have been infected with SARS-CoV-2, 3.8 million of whom have died, and these numbers continue to grow [1]. Vaccines for COVID-19 are critical tools for controlling the pandemic. In early December 2020, the Pfizer–BioNTech mRNA COVID-19 vaccine BNT162b2 received a temporary emergency use authorization in the United Kingdom, followed by a series of approvals or authorizations for emergency use in Bahrain, Canada, Mexico, Saudi Arabia, and the United States [2]. In Japan, BNT162b2 received a special approval for emergency use on 14 February 2021 [2]. The BNT162b2 mRNA vaccine is a lipid nanoparticle-formulated, nucleoside-modified RNA that encodes the SARS-CoV-2 spike, modified by two proline mutations to lock it in the prefusion conformation [3]. The BNT162b2 mRNA vaccine is administered intramuscularly (ideally into the deltoid area of the upper arm) in two 30 μg doses, 21 days apart [3]. In a phase 2/3 multinational randomized placebo-controlled efficacy clinical trial (NCT04368728), among 36,523 participants with no evidence of existing or prior SARS-CoV-2 infection, there were eight cases of COVID-19 with onset at ≥7 days after receiving the second dose of BNT162b2. In contrast, there were 162 cases of COVID-19 with onset at ≥7 days after receiving the second dose of placebo, corresponding to 95.0% vaccine efficacy (95% confidence interval, 90.3–97.6) [3]. The most frequent adverse effects reported after the administration of BNT162b2 were injection site pain, fever, fatigue, and headache, which occurred soon after vaccination and resolved shortly [3]. However, there is still a lack of data on the BNT162b2 mRNA vaccine for medium- to long-term safety, interaction with other drugs, and use in subjects with underlying medical conditions. Immune thrombocytopenia (ITP) after COVID-19 vaccination has been reported and is more common with the adenovirus vectored vaccine ChAdOx1 nCoV-19 (AstraZeneca) [4], but has also been reported with mRNA vaccines [5,6,7,8]. No other autoimmune diseases have been reported to be associated with mRNA COVID-19 vaccines. We report here the case of a patient who developed antineutrophil cytoplasmic antibody (ANCA)-associated vasculitis (AAV) after receiving the COVID-19 vaccine BNT162b.

## 2. Case

A 37-year-old Japanese woman had been taking propylthiouracil for Graves’ disease for about 28 months. She received the Pfizer–BioNTech COVID-19 mRNA vaccine BNT162b through her employment as a care worker. A small, round, and slightly raised erythema appeared on the forearm and precordium of the patient on the 12th day after she received the first dose of the vaccine, and a fever in the 37 °C range appeared on the 13th day. She visited a nearby hospital, underwent a blood test, and was prescribed a topical steroid. On the 25th day, pain, redness, and swelling appeared in the left auricle. Blood test results showed elevated levels of myeloperoxidase (MPO)-ANCA and proteinase 3 (PR3)-ANCA, which were negative before the patient had received treatment for Graves’ disease, suggesting the presence of propylthiouracil-induced AAV. On the 26th day after the first dose, a biopsy of an erythema on the right forearm was performed, and propylthiouracil was discontinued. The biopsy results showed infiltrations of lymphocyte-dominated inflammatory cells around the capillaries in the dermis and subcutaneous fat, but no fibrinoid necrosis or leukocytoclastic vasculitis characteristic of AAV. The patient’s erythema was alleviated, but a low-grade fever and swelling of the left auricle persisted. She was treated with garenoxacin mesilate hydrate for 5 days, but her symptoms did not improve. She was referred to our hospital on the 34th day after her vaccination for further examination and treatment and was admitted on the 35th day.

**Figure 1:**
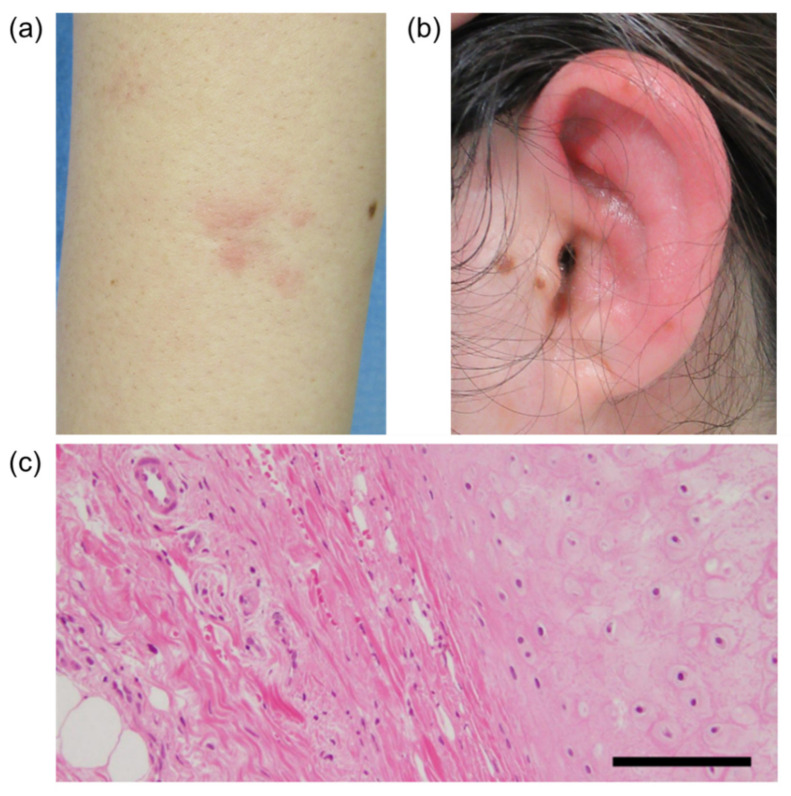
The patient’s right forearm (**a**) and left ear (**b**). (**c**) Pathological findings for the auricular cartilage. Hematoxylin and eosin (H&E) staining, original magnification 200×. Scale bar, 20 μm.

On admission, erythema was found on her bilateral forearms (Figure 1A), forehead, and thighs. The left auricle was red and swollen with tenderness (Figure 1B). The laboratory findings on admission are shown in Table 1. There were no major abnormalities in the complete blood cell count or biochemical tests except for an elevated C-reactive protein (CRP) level of 10.16 mg/dL (reference value < 0.14 mg/dL). The CRP level is usually elevated after immunization, and this is also true for BNT162b, but this CRP level was remarkably high and persisted even though it had been a long time since the patient’s vaccination. Serums MPO-ANCA and PR3-ANCA were increased to 494 IU/mL (reference value < 3.4 IU/mL), and 28.3 IU/mL (reference value < 1.9 IU/mL), respectively, and there were no abnormalities in other measured autoantibody tests including antinuclear antibody. Urinalysis showed no proteinuria or hematuria suggesting the presence of nephritis. Computed tomography of the whole body showed no obvious abnormalities, including thickening and narrowing of the trachea and bronchi. On the third day after admission, the erythema had almost disappeared. The redness and swelling of the left auricle persisted, and on the sixth day after admission, a biopsy of the left auricular cartilage was performed to differentiate relapsing polychondritis (RP). There was a mild lymphocytic infiltration in the dermis, but no inflammation or destruction of cartilage, which are typical features of RP (Figure 1C). Based on the patient’s history and blood and pathology results, we made the diagnosis of propylthiouracil-induced AAV.

On the ninth day after admission, prednisolone 30 mg/day was commenced. Her CRP levels quickly fell within the reference range and her left auricular symptoms gradually resolved. On the 23rd day after admission, the dose of prednisolone was reduced to 20 mg/day. She was discharged on the 24th day after admission.

## 3. Discussion

AAV is a group of disorders characterized by the inflammation and destruction of small- and medium-sized vessels and the presence of ANCA [9]. The spectrum of AAV includes granulomatosis with polyangiitis (GPA, Wegener’s granulomatosis), microscopic polyangiitis (MPA), and eosinophilic granulomatosis with polyangiitis (EGPA, Churg–Strauss syndrome). AAV can result in damage to various organs including the respiratory system, kidneys, eyes, skin, and nervous system. In some patients, drug-induced AAV occurs as a complication after the use of certain therapeutic agents, and propylthiouracil is the most frequently reported causative agent [10]. The pathogenesis of propylthiouracil-induced AAV has not been fully elucidated. The interactions between propylthiouracil and the target antigens of ANCA, especially MPO, may be involved in the development of propylthiouracil-induced AAV [11].

RP is a rare autoimmune inflammatory disease characterized by recurrent inflammation and destruction of the cartilage in various sites of the body, including the auricle, nose, throat, trachea, and bronchi [12]. Auricular chondritis is the most frequent presenting manifestation in patients with RP. Even in RP, ANCA may occasionally be positive [13], and one case report described the development of ANCA-positive RP in a patient being treated with propylthiouracil [14]. The diagnosis of RP is based on typical clinical findings. According to McAdam et al., RP can be diagnosed if three or more of six clinical features are present: bilateral auricular chondritis, nonerosive inflammatory polyarthritis, nasal chondritis, ocular inflammation, respiratory tract chondritis, and audiovestibular damage [12]. A cartilage biopsy is not essential for diagnosis but may contribute to the diagnosis in cases of atypical clinical presentation [15,16]. In the present case, the patient had unilateral auricular symptoms but no other typical RP findings, and an auricular cartilage biopsy showed no cartilage inflammation. Given the results of the skin biopsy, the main inflammation in this case seemed to be of small blood vessels rather than cartilage. Therefore, although RP could not be ruled out, we diagnosed this case as AAV, and believe that the AAV caused RP-like auricular inflammation.

The standard remission induction regimen for primary AAV is the combination of high-dose glucocorticoids and cyclophosphamide or rituximab [17]. Since the pathogenesis of primary and drug-induced AAV is different, there is no standard treatment strategy for drug-induced AAV [10]. It is important to discontinue the causative drug immediately after the diagnosis of drug-induced AAV. The prognosis of drug-induced AAV is considered to be better than that of primary AAV [10]. Once the causative drug is discontinued and the disease goes into remission, relapse is rare and maintenance therapy may not be necessary [10]. In the present case, glucocorticoids were administered because discontinuation of the causative drug, propylthiouracil, did not sufficiently improve symptoms. The patient’s symptoms improved after treatment with moderate doses of prednisolone.

There are several reports of new-onset AAV following COVID-19 [18,19,20]. Direct endothelial injury by the virus, complement activation, and activation of neutrophils resulting in the formation of neutrophil extracellular traps (NETs) may drive the development of AAV [21,22]. However, the relationship between AAV pathogenesis and COVID-19 vaccines is not known, and the present case provides novel aspects in AAV pathogenesis.

The safety profile of BNT162b is characterized by injection site pain, fever, fatigue, and headache, with no reports of vasculitis [3]. Since the vaccine was developed urgently, no long-term studies have been conducted. There are still concerns about unknown long-term adverse effects of the vaccine [23]. Several cases of thrombocytopenia have been reported after the administration of mRNA vaccines [5,6,7,8]. In a report from the U.S. of 20 patients who developed thrombocytopenia after vaccination, nine patients had received the Pfizer–BioNTech vaccine and 11 received the Moderna vaccine [6]. All 20 patients were hospitalized and most of them presented with petechiae, bruising, or mucosal bleeding with the onset of symptoms between 1–23 days (median 5 days) after vaccination. The majority of patients’ platelet counts at presentation were ≤10 × 10^9^/L (range 1–36 × 10^9^/L; median 2 × 10^9^/L). Most of those patients were successfully treated with corticosteroids and intravenous immunoglobulin (IVIG), suggesting that their thrombocytopenia may have been due to secondary immune thrombocytopenia (ITP) after mRNA vaccination. Each year in the U.S., approx. 50,000 adults are diagnosed with ITP. Given the size of the vaccinated population, the incidence of ITP after vaccination is probably not higher than the incidence of cases that occur otherwise [6]. It is unclear whether these cases are secondary ITP caused by vaccination. Although the cases are rather rare, the potential of the Pfizer–BioNTech and Moderna vaccines to ITP cannot be ruled out and requires continued surveillance.

Other than ITP, there have been no reports of autoimmune diseases developing after COVID-19 vaccination. Adverse reactions to the vaccines are thought to be the result of the interaction between the susceptibility of the vaccinated subject and various vaccine components. Similarities between certain pathogenic elements contained in a vaccine and specific human proteins may be associated with adverse immune responses to the vaccine [24]. This similarity may lead to immune cross-reactivity, and the reaction of the immune system to the pathogenic antigens may cause autoimmune diseases [24,25].

It has been reported that there is homology between the nucleoprotein/spike protein of SARS-CoV-2 and human tissues [25]. The immune response to the SARS-CoV-2 antigens following infection or vaccination may cross-react with human tissue antigens that share sequence homology with the virus and may lead to the development of autoimmune diseases in the long-term [26,27]. Talotta noted that in patients with immunological and serological abnormalities in the absence of clinical symptoms, the administration of the vaccine may put these patients at risk of unwanted immunological side effects by sensitizing pattern recognition receptors (PRRs) or generating cross-reactive cell clones or antibodies [27].

In our patient’s case, propylthiouracil is considered to be the main cause of the onset of AAV. It has been reported that MPO-ANCA was detected in 37.5% of patients with Graves’ disease being treated with propylthiouracil [28]. Even if ANCA is induced by propylthiouracil, most of the patients do not develop AAV. However, some patients developed AAV at a median of 39 months after propylthiouracil administration [29]. In the present case, we speculate that the patient was already positive for ANCA immediately before the vaccination. We believe that the patient became ANCA-positive due to prolonged propylthiouracil treatment, which subsequently led to the development of AAV. Although the impact of the BNT162b2 vaccine on this case is unknown, we cannot rule out the possibility that the vaccine may have triggered the onset of AAV in the patient, who is thought to have been in the precursor condition of developing the disease.

To the best of our knowledge, this is the first report of a case of propylthiouracil-induced AAV that developed after vaccination with BNT162b2. Although the development of AAV in this patient may have been incidental and unrelated to the vaccination, this report provides important data for evaluating the safety of the vaccine. BNT162b has been documented to have enormous utility and adequate safety benefits. The benefits of the protection offered by BNT162b outweigh the risk of rare potential side effects. We emphasize that vaccination with BNT162b should not be avoided because of this very rare event. Careful safety monitoring should be continued to assess the potential risk of the vaccine.

## 4. Conclusions

This is the first report of propylthiouracil-induced AAV developing after COVID-19 vaccination. Although the onset of disease may have been accidental and unrelated to the patient’s vaccination, this report is important for scrutinizing and evaluating the safety of the COVID-19 vaccine.

## Figures and Tables

**Table 1:** The patient’s laboratory test results on admission.

**Complete blood counts:**			Total bilirubin	0.5	mg/dL
White blood cells	7490	/μL	Aspartate transaminase	12	U/L
Neutrophils	69.2	%	Alanine aminotransferase	13	U/L
Lymphocytes	21.6	%	Lactate dehydrogenase	127	U/L
Red blood cells	434 × 10^4^	/μL	Creatinine kinase	13	U/L
Hemoglobin	12.7	g/dL	TSH	2.46	μIU/mL
Hematocrit	38.4	%	Free thyroxine	0.99	ng/dL
Platelets	46.7 × 10^4^	/μL	**Immunology:**		
Urinalysis:			Rheumatoid factor	negative	
Protein	22	mg/dL	Antinuclear antibody	negative	
Creatinine	239.2	mg/dL	Anti-ds-DNA antibody	negative	
Estimated urine protein	0.1	g/day	Anti-GBM antibody	negative	
Biochemistry:			PR3-ANCA	28.3	U/mL
C-reactive protein	10.16	mg/dL	MPO-ANCA	494	U/mL
Blood urea nitrogen	9.5	mg/dL	CH50	83	U/mL
Creatinine	0.62	mg/dL	IgG	1366	mg/dL
Total protein	8.1	g/dL	IgA	245	mg/dL
Albumin	4	g/dL	IgM	247	mg/dL

## Data Availability

The data presented in this study are available on request from the corresponding author.
